# Supplementary material for: Parasitism by the Tachinid Parasitoid Exorista japonica Leads to Suppression of Basal Metabolism and Activation of Immune Response in the Host Bombyx mori
Source: Insects. 2022 Aug 31;13(9):792. doi: 10.3390/insects13090792 (PMC9506100; doi:10.3390/insects13090792)
Supplement: Supplementary file 1 [file insects-13-00792-s001.zip › insects-1866849-supplementary.pdf]

Table S1. List of RT-qPCR primers.

Gene ID	Gene	Forward primer	Reverse primer
BMSK0009350	*PGRP-LB*	CGGCCCACAAGATTATCGGA	AATGGAACCCGGGAAAGGTG
BMSK0003373	*ATG16L1*	GCAACCCTACCGCGTTTTTC	TCTTGTCGTCTGTGCTTCCC
BMSK0015671	*HSP70*	GGACACTCTCATCCAGCACC	CTCGACGGGTTCCAGAGTTC
BMSK0007592	*cytochrome b-c1*	TGCGTACTGGTTACCACCTG	AGTCCCTTGGGTCTTTTCTCA
BMSK0009526	*FASN*	CGGTATCAGCGATGTGTCCA	TTTAGCTGCCAACTCTGCGA
BMSK0007257	*Mdh*	AATGTTCCCAGGTCGCAAGT	GGAAAGGTGTCCTTGTCCGT
BMSK0003612	*Gdh*	GCATGCTGCAAAATCCCACA	CAGAAAGCCAAGCCGTGTTC
BMSK0007924	*Atifm1*	TCGAACGTAAGCCCGATGAG	GCGTCGTACACTTGCGTTTT
BMSK0014004	*Hemolin*	TAGTGTCGGTCAAGACACGC	AAGCACTGGTACTCGCCTTC
BMSK0005301	*hemocytin*	CCGAGTGTCCTTGTGTCGAA	GGTGGTCCCTCCTAGTTTGC
NM_001043611.1	*CTL11*	GAAGCAATACCGCTCCGACT	CTCCCTCAGTGGTGCAAGAG
BMSK0016016	*Gloverin1*	GGCTGCTATTGACTTGAAC	TCTGTGACCGAACTCCTT
BMSK0009812	*Gloverin2*	ACAAGCCACTATTGACCTAA	TTGTGACCGAACTCCTTC
BMSK0013244	*Moricin*	GCAATGTCTCTGGTGTCA	GCGATATTGATGGCTCTTAG
BMSK0013249	*MoricinB3*	ATGCTCGTCCTCATAATGG	AATAACTTTCCCGCCCTTT
BMSK0003513	*cecropin A*	CTCCTGAGCCCAGGTGGAAAC	AAGGCAATGACTGTGGTATG
BMSK0007319	*Cactus*	CCTCCGCTGTATCTACTGTTCC	ATGGCATTACCACTCATCACC
BMSK0005167	*Spatzle*	AGCGTAAGTCAGTCGCCAAA	GAGAGTCGTCCGTTTGAGCA
NM_001102465.1	*Relish*	GAACAGCCACAGGACTACTTTA	ATGATACCCATTCCACCGAAC
MF374784	*Imd*	AGCGCCTTTGAAGCCAATGT	TTTCGCCTGTGGCTACTCCA
BMSK0014150	*Socs6*	GCAGTCACCATCAACATGCA	GCCAGCAAGCAACTCTTCTG
BMSK0005942	*STAT*	GAGCGTTATGGACGAGAAGC	CCTGGTTGCCGTGGACTATG
BMSK0011178	*Hop*	CTACCGGCTACGTATGGTGC	TTCGGAGACGCATAAGCACA
BMSK0009998	*RpSA*	GATGTGGTGGTTGTTGGCAC	GCCTGTTCCTTGGCTTGTTG
BMSK0008977	*RpP0*	GGGTAGGGAGGACAAGGCTA	GGAGCCACGTAGCGAGATAC
BMSK0013501	*Bcp*	CCGAGCAGTCGATGAGGTTT	CCAAGGGAAGTGTGGCTCAT
BMSK0002236	*Scp*	AGAACGCGAATGGACTTGGT	TACAGATCCGAAGCGCACTC
BMSK0011786	*Pgi*	ATTTCATCGCTCCAGCCCAA	CCTTCATCAGGGCTTCGGTT

BMSK0002731	*Pfk*	GTCTTCATTGGCTCGTTGAG	AGGATACGCCGCAATGGACG
BMSK0000274	*Tpi*	ACCCGCTATGATCAAAGACA	CAACTTCCTCAGTCTTGCCA
BMSK0001249	*Gadph*	AGTATGATTCCACCCATGGC	TCCCTCCAAGTGAGCAGATG
BMSK0009616	*Pglym*	CTTCTGTGGATGGTTCGACG	TGCCTCTCGTTCAATCTCCA
BMSK0004515	*Eno*	ACAAACCCTAAGCGTATCG	AACTACCAGGTCGGCAATA
BMSK0000579	*Ldh*	ACTAACTTGGACTCGGCACG	GACCATACTGGAACGCTGCT
BMSK0007255	*PLCB1*	ATGCTAAAGTGCCAAAAG	AAGCCAACGCAAACAAC
BMSK0002015	*DGAT1*	CCCTTTGCTATGAGTTGA	AGACAGAGCCAGATGAGAT
BMSK0007427	*PLD2*	GCCGTCACGCACTGGA	AGCGGTCGTTGAGGTT
BMSK0002871	*ACER3*	GTTCTTTCTGTTCCCACC	TGCTGCCATAAATACCC
BMSK0014837	*CRLS1*	CTAATAGTTGGGCGAGAT	CACAAGACTTTCAATGCTG
BMSK0012852	*SGMS1*	ATTGGGGTAACAAAGAAGT	ACAAACGATAGCAGGCAC
BMSK0002725	*SPH*	GGAAACCACGAATCTCA	CGTCCAAGGGGTCATA
BMSK0009186	*CERS3*	CACCAATAGCACTCCGTC	CCATCTCCTGCCTTTCCT

Table S2. The data filter of RNA-seq and the mapping results.

Category	Parameter	NP	P
Data filter	Total raw reads (M)	54452236	56018444
	Total clean reads (M)	44977426	46762661
	Total clean base (Gb)	6680945135	6979446638
	Clean reads Q20 (%)	98.07	98.15
	Clean reads Q30 (%)	95.15	95.36
Mapping to genome	Total clean reads (M)	44977426	46762661
	Maped-rate (%)	90.56	91.26
